# Surgical treatment using The Unit Rod in children with neuromuscular scoliosis

**Published:** 2016

**Authors:** T Nedelcu, I Georgescu, J Leroux, J Lechevallier, A Barbilian, I Tuhar

**Affiliations:** * Paediatric Surgery Department, Le Havre General Hospital, France; ** Paediatric Orthopaedics Department, Paediatric Physical Rehabilitation Centre Bois Larris, Lamorlaye, France; *** Paediatric Surgery Department, Rouen University Hospital, France,; **** Orthopaedics and Traumatology Department, “Carol Davila” University Emergency Military Hospital, Bucharest, Romania; ***** Department of Plastic Surgery, “Carol Davila” University Emergency Military Hospital, Bucharest, Romania

**Keywords:** neuro-muscular scoliosis, cerebral palsy, vertebral arthrodesis, Unit Rod, sublaminar wires

## Abstract

The article represents a retrospective clinical and radiological study.

**Objective.** To assess the safety and the stability in time of the Unit Rod instrumentation in the treatment of severe neuromuscular scoliosis in children and adolescents.

**Summary.** The treatment of patients with neuromuscular scoliosis always represents a challenge. The patients are debilitated and usual interventions are very long with great loss of blood. Serious complications can compromise the result of the surgery. The technique we used (the Unit Rod) is worldwide recognized, is simple, and gives excellent stability with a low rate of complications.

**Methods.** We conducted a clinical and radiological retrospective study with a follow-up of at least 4 years in 58 patients with serious neuromuscular conditions, most of them being non-walkers. They were surgically treated by using mostly the Unit Rod technique, in the department of Paediatric Orthopaedics of the Rouen University Hospital, France, between 2000 and 2008. The back fusion was generally from T2 to pelvis. We used the Galveston technique for the patients who needed a pelvic fixation.

**Results.** The mean Cobb angle correction was of 67% immediately after surgery; the correction of the curve decreased in time only in 4% of the cases. Pelvic obliquity was also very well corrected: 73% immediately and 70% at the last radiological follow-up. The mean operative time was of 175 minutes compared to 269 minutes for screws and hooks instrumentation. The most common complication for our technique was the radiolucent halo that appeared around the pelvic inserts. There was no significant degradation in time of the correction obtained.

**Conclusions.** The use of this technique is safe, gives excellent results, achieving significant improvements in the postoperative functional status of the patients. The intra- and postoperative complications were minor. The advantage of using this method is the low cost of the material and technical simplicity, the corrective results being the same as the ones obtained with other techniques.

## Introduction

Management of neuromuscular scoliosis, ranks second in frequency of all scoliosis, encounters some of the most difficult problems, being a controversial subject in paediatric orthopaedic surgery. Conservative treatment is generally ineffective, being difficult to tolerate in this type of patients [**1**]. Surgery is very effective but comes with a price: high rate of complications and also high costs including the effective cost of the implants and the long hospital stay (with at least one week in the intensive care unit). The management of this type of scoliosis is difficult for spine surgeons but also for the para-medical and nursing staff involved in the rehabilitation of these patients. Despite all those difficulties, surgery alone can reduce morbidity and mortality of the neuro-muscular scoliosis patients.

Neuro-muscular scoliosis is actually a “symptom” encountered in patients suffering from various neuromuscular disorders: cerebral palsy, myelomeningocele, spinal cord injury, muscular dystrophy, spinal muscular atrophy, etc. Despite the large etiological differences, all the neuromuscular pathologies show a common feature: trunk hypotonia or muscular imbalance. Paravertebral muscles, affected by the disease, cannot provide sufficient support to maintain an accurate static of the trunk [**2**]. Usual curves that occur in the neurological or muscular diseases have similar physiopathological mechanisms and evolution patterns. Neuromuscular scoliosis is generally a long thoracolumbar “C” curve [**3**].

A common feature for patients with neuromuscular scoliosis is that this frontal curve is often associated with kyphosis (inverse situation encountered in the case of idiopathic scoliosis). Once structured, this type of scoliosis is often progressive. This progression depends heavily on the aetiology, age of onset of the disease and the severity of the curve. However, unlike idiopathic curves, the neuromuscular ones could be progressive even after the completion of growth [**4**].

Due to the wide variety of the aetiology, the incidence of neuromuscular scoliosis varies widely (**Table 1** shows this incidence as presented by the Scoliosis Research Society). It is known that the incidence of scoliosis in neuromuscular patients is higher than in the general population, that the usual evolution is towards aggravation even after growth finishes, and that the only pertinent treatment is surgery. Among patients with neuromuscular diseases, the likelihood to develop scoliosis is inversely proportional to the ambulatory ability. Thus, the risk of scoliosis in non-ambulatory patients, regardless of the underlying aetiology, is of 80-90%.

**Table 1:** Incidence of scoliosis depending on aetiology

Pathology	Incidence
Cerebral Palsy	
GMFCS I and II (ambulatory)	25%
GMFCS III and IV (non-ambulatory)	80-90%
Neuropathies (Charcot-Marie-Tooth)	30%
Lumbar syringomyelia	60%
Thoracic syringomyelia	100%
Spinal muscular atrophy	70%
Friedreich’s ataxia	80%
Duchenne muscular dystrophy	90%
Spinal cord injuries	100%
*GMFCS - Gross Motor Function Classification System for Cerebral Palsy	

Nowadays, there is a consensus that the only effective treatment for neuromuscular scoliosis is the spinal fusion. Therefore, as soon as the curve reaches a certain threshold, surgery should be considered. This threshold varies with the pathology. However, in muscular dystrophies the trend is to operate quite early, as soon as the Cobb angle reaches 30°, in the case of cerebral palsy or myelomeningocele we can usually wait for the curve to reach 50° [**5**].

The lower level of the fusion remains controversial, the pelvic fusion being generally required. Usually, this fusion is not recommended for walking patients. Some authors still prefer to fuse even the non-walkers to L5 [**6**] because the pelvic instrumentation is difficult, it increases the surgery time, the bleeding, as well as the rate of complications. The concept sustained by most of the spine surgeons is to fuse the pelvis if the pelvic obliquity exceeds 15° [**7**,**8**].

For non-ambulatory patients, the arthrodesis’ purpose is to obtain a straight spine, perpendicular to a balanced pelvis, and so to insure the sitting position. Previous studies showed that if the pelvic obliquity is not corrected, the main purpose of arthrodesis is not reached [**9**]. Those patients usually show a lumbar hyperlordosis, which is usually corrected by using a pelvic fixation with the Unit Rod [**10**].

Surgery is proposed to patients who have rapidly progressive curves that can have a negative impact on their quality of life (respiratory function, posture, social integration). Generally, a single posterior approach is needed for these patients. There are studies that have shown a good stability of the correction without inducing the “Crankshaft” phenomenon with this single posterior approach, using the Unit Rod, even for skeletally immature patients (triradiate cartilage still open). Perhaps the solidity of this construct, anchored in the pelvic grid, prevents vertebral rotation even if the anterior epiphysiodesis of the vertebral bodies is not performed [**11**,**12**].

There are two main surgical techniques that can provide spinal fusion for neuromuscular scoliosis: segmental instrumentation with sublaminar wires (Luque) plus Unit Rod inserted in the pelvis as described by Galveston [**13**,**14**], and the segmental screws and hooks developed by Cotrel-Dubousset [**15**,**16**]. There are authors who use a combination of this two techniques (hybrid construct), using especially lumbar screws to improve solidity.

Unit Rod instrumentation is used for spinal deformities requiring arthrodesis to the pelvis. It is used in non-ambulatory patients [**17**,**18**]. Some authors described it also for walking patients; in those cases, the anterior-posterior orientation of the pelvis should be carefully assessed pre-operatively, because changing this orientation could lead to the loss of ambulation. This method saves time, diminishing intra-operative complications, and the rate of further surgery. Beside the medical advantages stated above, one of the arguments in favor of this technique is the low cost of this instrumentation. Until now, this technique has been regarded as the treatment of choice for neuro-muscular scoliosis [**18**].

## Materials and methods

We retrospectively analyzed clinical and radiological data of 58 patients with neuromuscular scoliosis operated by the same surgeon in the Department of Paediatric Surgery in Rouen University Hospital, France, between 2000 and 2008. Out of these 58 patients, 42 were operated by using the Unit Rod technique and 16 were operated by segmental instrumentation with screws and hooks. Out of these last 16, eight patients were walkers and the pelvis did not need any instrumentation, and the other eight were non-walkers and the pelvis was fused by using sacral screws. Average follow-up was at 6.7 years (range 4 to 13 years). 38 boys and 20 girls were included in the study (**Table 2** shows the demographics of patients included in the study). In terms of the aetiology, the most common pathology was represented by cerebral palsy (CP) - 22 patients, followed by Duchenne muscular dystrophy (DMD) - 13 patients (**Table 2** shows the aetiology of scoliosis in our patients).

**Table 2:** Patients’ aetiology and demographics

Name	Sex	Birthdate	Disease	Walker/ Non-walker W/ NW	Year of the intervention	Age at the operation (years, months)	Follow-up (Years)
A.N.	M	10/06/1985	Friedreich ’s Ataxia	W	2002	17+2	6
A.E.	M	25/12/1985	Cerebral Palsy	NW	2000	14+10	8
A.E.	F	29/07/1996	RETT's Syndrome	NW	2007	11+6	8
A.G.	M	09/09.1987	Traumatic spinal cord injury	NW	2000	13+2	11
A.M.	M	13/07/1992	Metabolic disease: Juvenile Ceroid lipofuscinosis	NW	2006	14+1	4
A.M/	F	19/01/1994	Spinal muscular atrophy type II	NW	2006	12+6	5
B.T.	M	20/08/1992	Cerebral Palsy	NW	2006	15+10	4
B.S.	F	01/09/1992	Arthrogryposis	W	2005	13+0	7
B.M.	M	14/02/1990	Cerebral Palsy	NW	2006	15+9	5
B.J.	M	28/08/1989	Duchenne muscular dystrophy	NW	2004	15+0	10
B.V.	M	15/11/1994	Duchenne muscular dystrophy	NW	2008	13+4	5
B.O.	M	27/11/1980	Cerebral Palsy	W	2006	26+1	6
B.I.	F	03/11/1984	Congenital myopathy (merozyne deficit)	NW	2005	10+5	9
B.M.	F	17/07/1989	Cerebral Palsy	NW	2004	15+6	4
B.B.	M	23/05/1992	Duchenne muscular dystrophy	NW	2004	11+11	5
C.J.	F	14/01/1984	Unknown genetic syndrome	NW	2004	20+0	4
D.T.	M	02/01/1987	Cerebral Palsy	NW	2003	16+8	4
D.E.	F	30/04/1991	Unknown genetic syndrome	W	2003	12+1	5
D.P.	M	19/10/1987	Cerebral Palsy	NW	2004	16+8	4
D.V.	M	13/10/1987	Alcoholic fetopathy	W	2001	13+10	9
D.Q.	M	06/10/1993	Duchenne muscular dystrophy	NW	2005	11+7	8
D.H.	M	16/06/1992	Unknown genetic syndrome	NW	2008	15+8	6
D.E.	F	22/04/1985	Arthrogryposis	NW	2000	15+2	11
D.C.	F	03/02/1987	Cerebral Palsy	NW	2002	14+0	5
F.A.	M	13/05/1993	Cerebral Palsy	NW	2006	13+4	8
F.G.	M	16/10/1991	Duchenne muscular dystrophy	NW	2006	15+0	8
F.R.	M	02/08/1988	Duchenne muscular dystrophy	NW	2002	13+4	6
G.N.	M	22/10/1990	Duchenne muscular dystrophy	NW	2002	11+7	4
G.P.	F	20/08/1987	Cerebral Palsy	NW	2006	18+10	4
H.A.	M	05/08/1984	Cerebral Palsy	NW	2004	20+1	4
H.B.	M	20/04/1990	Duchenne muscular dystrophy	NW	2003	12+10	5
H.J.	M	26/12/1988	Cerebral Palsy	NW	2004	15+6	6
H.K.	F	30/07/1985	Cerebral Palsy	NW	2006	20+10	7
I.A.	F	09/07/1993	Congenital Nemalin myopathy	NW	2005	12+6	9
K.H.	M	10/12/1985	Cerebral Palsy	NW	2000	15+0	7
L.C.	F	16/05/1986	Cerebral Palsy	NW	2001	15+3	5
L.L.	M	04/06/1997	Duchenne muscular dystrophy	NW	2008	11+2	6
L.A.	M	11/07/1986	Cerebral Palsy	W	2000	14+0	4
L.K.	M	17/06/1988	Arthrogryposis	NW	2004	16+5	4
L.F.	M	02/09/1984	Cerebral Palsy	NW	2001	16+2	4
L.J.	M	13/07/1993	Duchenne muscular dystrophy	NW	2006	12+10	7
L.K.	F	19/12/1985	Cerebral Palsy	NW	2001	15+11	13
M.N.	M	26/10/1980	Cerebral Palsy	NW	2002	21+1	8
M.A.	M	20/07/1992	Duchenne muscular dystrophy	NW	2003	11+3	9
M.D.	M	01/07/1989	Syringomyelia type I	W	2006	17+2	7
M.N.	M	19/11/1981	Sensitive motor neuropathy (Charcot Marie Tooth)	W	2001	20+1	10
M.A.	F	26/05/1984	Cerebral palsy	NW	2002	17+10	12
M.F.	M	15/06/1988	Duchenne muscular dystrophy	NW	2002	13+9	10
N.M.	F	28/11/1984	Unknown genetic syndrome	NW	2003	18+10	5
O.D.	M	29/02/1984	Cerebral Palsy	NW	2001	17+9	6
P.D.	F	19/05/1992	Medullary tumor	NW	2004	12+0	10
Q.F.	M	04/10/1992	Cerebral Palsy	NW	2008	15+1	6
R.V.	M	07/08/1989	Duchenne muscular dystrophy	NW	2002	15+9	7
R.E.	F	24/06/1991	RETT's Syndrome	NW	2003	11+7	8
R.L.	F	24/06/1991	RETT's Syndrome	NW	2004	12+10	7
V.B.	M	04/11/1989	Sensitive motor neuropathy (Charcot Marie Tooth)	W	2006	16+9	4
V.D.	F	09/07/1984	RETT's Syndrome	NW	2002	17+10	6
W.T.	M	10/10/1989	Encephalitis	NW	2004	15+1	10

The inclusion criteria for the study were (for all 58 patients): neuro-muscular disease at the origin of the scoliosis diagnosed by a paediatric neurologist, scoliosis diagnosed clinically and radiologically. They were all operated by two techniques (Unit Rod and segmental screws and hooks) by the same surgeon in the Department of Paediatric Orthopaedics in Rouen. The follow-up period was of minimum 4 years. Complete clinical and radiological files for all the patients were available.

The surgical indication was established by assessing the radiological factors: type of curve and its progression pattern. Important clinical elements taken also into account were diminishing tolerance of the sitting position in a wheelchair, sagittal or frontal imbalance of the trunk, tendency to pressure sores, respiratory compromised condition, and lack of response to a possible orthopaedic treatment. Another clinical factor for the choice of the surgical technique was the walking ability of the patients. The main surgical technique used for most of the patients studied was the Unit Rod. It was the choice technique for those patients in the Paediatric surgery Department of Rouen. Spinal fusion was performed between T2 and pelvis.

**Surgical technique**

Every level from T2 to L5 needed a sublaminar wire at each side. These wires were inserted after the liberation of the yellow ligament between two adjacent laminas. Luque described the original technique for the instrumentation of idiopathic scoliosis. It was the first segmental technique. He used two separate rods, without sagittal bending. The rods were inserted para-vertebral and the wires tightened while gradually bringing the spine to the rod. For neuro-muscular scoliosis, this technique needed improvement so that the pelvis could be included in the construct. At that moment, Galveston [**13**] developed pre-bend rods (at 90°) so that the horizontal part could be inserted in the iliac wing.

**Fig. 1:**
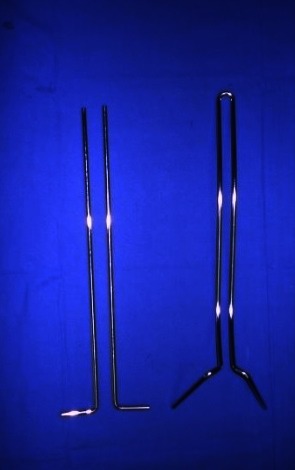
Initial Luque-Galveston rods vs. Unit Rod

The Unit Rod is a unique double rod, proximally united, pre-bent to respect sagittal plane, with two pelvic insertions at around 45° from the direction of the rod. The entry point of the pelvic insertions is immediately distal to the posterior iliac spines. The direction of the rods is 1-2 cm proximal from the ischial notch. Penetration must be between 6 and 10 cm. Originally, two independent rods were used (**Fig. 1**). Because of the lack of the unity between those separate rods, in immature patients, the crankshaft phenomenon was not avoided.

First, 2 sublaminar wires were implanted at every level from T2 to L5. We used two wires for each side for T2, this level being subjected to higher tensile strengths and wire breakage.

By reinforcing this level, we avoided postoperative complications. After the preparation of the spine, we perforated the tunnel in the iliac wing with a special guide. The Rod was then inserted with its two endings in the tunnels. At that moment, with the strength of the rod we were able to diminish the pelvic obliquity and pass the wires around the rods. We tightened the wires progressively so that the spine was perpendicular to the pelvis and the scoliotic curve was reduced. At the end we put the morselized graft obtained from the spinal and transverse apophysis from T2 to L5 in place around the rods.

**Fig. 2:**
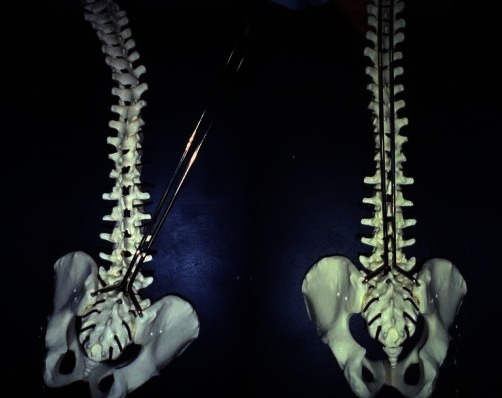
Insertion of the rod in the pelvis. Correction of the pelvic obliquity by bringing the rod to the spine

The major coronal curve was measured on A-P radiographies by Cobb’s method. Secondary curves were omitted from the analysis. Preoperative bending radiographs were also evaluated. Cobb angle reduction on these radiographs was used pre-operatively to assess the expected surgical correction. Pelvic obliquity was also calculated on A-P radiographies as the angle between the line tangential to the two iliac crests and a horizontal line.

## Results

The average follow-up was at 6.7 years with a range between 4 and 13 years. All 58 patients in the study met the inclusion criteria, having a comprehensive medical record, preoperative, immediate post-operative and at the last follow-up X-rays. **Table 3** shows the average postoperative Cobb angle and pelvic obliquity correction.

The mean age of patients on the date of surgery was 14.9 years (11-26 years). The mean age was lower for the patients with Duchenne muscular dystrophy (12.7 years) than for other neurological diseases, being conditioned by the respiratory and cardiac functions of these patients.

Postoperative outcomes assessment took into account the length of the surgery, the intra-operative bleeding -estimated only by the need of intra-operative transfusion, the intra and postoperative complications, the radiographic dynamics and the patient’s clinical status.

**Radiological results**

Cobb angle correction was of 67% immediately post-surgery and 63.7% at the last check-up (more than 4 years post- surgery). Initial mean Cobb angle was 63° (range between 25°-122°) which decreased to 20.7° after surgery (range 0° - 65°) and 22.9° at the last radiological check-up (range 0° - 70°). There were no differences or the Cobb angle correction obtained by the two techniques.

**Fig. 3:**
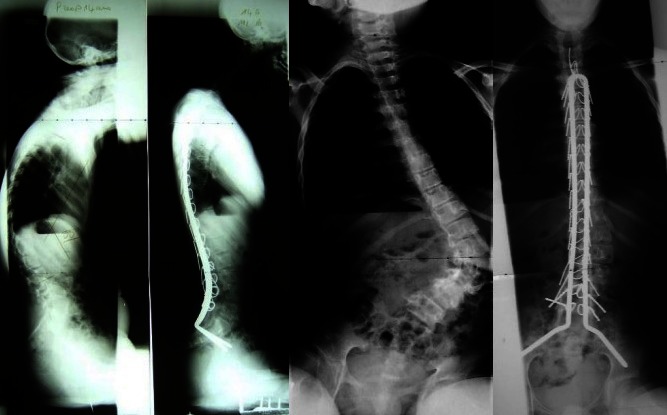
Pre and postoperative X-Rays for a Cerebral Palsy patient surgically treated by the Unit Rod method

For pelvic obliquity, the correction was of 72.8% immediately post-surgery and of 70.2% at the last check-up for patients operated with the Unit Rod. For patients operated with segmental fixation with screws and hooks, the pelvic obliquity correction was slightly lower, of 71.5% immediately after surgery and of 69% at the last check-up (**Table 3**). This difference taking into account the low number of patients operated by this last technique is not significant.

**Table 3:** Dynamic evaluation of radiological parameters

Radiological parameters	Pre-operatively	Post operatively	
		Immediate	> 4 years
Cobb angle (°)	63 (20-122)	20.8 (0-65)	22.9 (0-70)
Pelvic obliquity (°)	25.2 (0-53)	4.3 (0-20)	4.9 (0-21)

An important factor limiting the postoperative infection and other intra-operative complications was the surgery time. The average operative time was 175 minutes (range between 115 and 240 minutes) for patients operated with the Unit Rod technique. The operative time was a lot higher for the patients operated by the Cotrel - Dubousset technique, even if the pelvis was not instrumented: this technique takes a mean time of 269 minutes for a single posterior approach and of 340 minutes for anterior and posterior approach (**Table 4**).

**Table 4:** Mean surgery time

	Unit Rod	Segmental CD*	
Mean surgery time (minutes)	175 (115-240)	AVP 269 (200-320)	AVA+AVP 340 (300-420)
*CD – Cotrel – Dubousset			

Intra-operative bleeding was assessed only by the need for transfusion. 28 out of the 58 patients needed an intra-operative transfusion (48%). The bleeding rate was a bit higher for Duchenne patients who required transfusion in 54% of cases. In the surgical records, only two patients with Duchenne dystrophy presented excessive intra-operative bleeding.

The intra-operative transfusions were decided by the anesthetist in accordance with the intra-operative decrease in the haemoglobin level under the limit allowed by the department’s protocol (transfusion is considered necessary intra-operatively when Hb ≤ 9 g/ l and postoperatively when Hb ≤ 8g/ l).

Clinical improvement in patients was assessed by the possibility of a simple wheelchair installation.

**Fig. 4:**
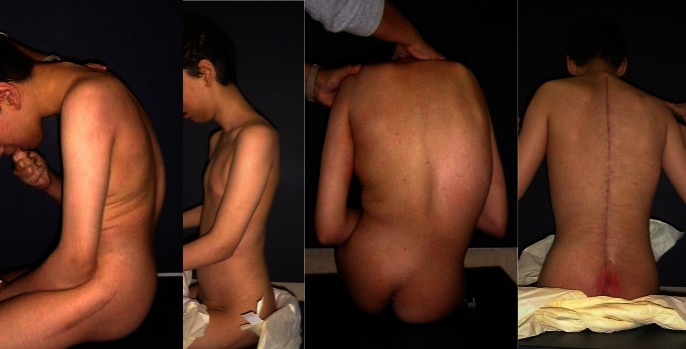
Pre and postoperative photographs of the same patient surgically treated by the Unit Rod method

The sitting balance of his pelvis changed a lot after the operation and the pressure sores disappeared (without brace stabilization), absence of ischial pressure sores improved the general status and social interaction. This information was obtained mainly through discussions with caregivers. Patients/ caregivers described: occasional pain, but with an obvious improvement of their general status compared to their pre-operative condition, an improvement in the comfort of life due to the lack of containment of the braces, decreased frequency of pressure sores (probably due to the improved balance of the pelvis), improved tolerance of the wheelchair, and better social integration.

During the surgery, the complications were minimal and without significant consequences in the post- operative period. They were represented by a false path in the iliac wing for the rod stem, which was solved by changing the inclination of the guide and creating a new access path at this level (7 patients); intra-operative abundant bleeding (2 patients, both with Duchenne myopathy); pleural breach (1 patient); epidural breach (1 patient); L5 posterior arch fracture (1 patient).

Postoperative complications: difficulties in scarring/ wound disunion (8 patients); postoperative infection (6 patients) - these patients were re-operated: debridement and surgical lavage were practiced and intra-venous antibiotic treatment according to the department protocol, but they did not require removal of the osteosynthesis material; proximal sublaminar wire breakage - T2 (4 patients) without secondary loss of correction; rod breakage and pseudarthrosis (2 patients) - these patients were re-operated and a new rod was placed; pulmonary complications (5 patients); postoperative pressure sores (2 patients); oversized material that produced skin conflict (1 patient) - this patient was re-operated to shorten the synthesis material; protrusion of the pelvic stem out of the iliac wing in the inner pelvis with pressure on the urinary bladder (1 patient ) - necessitated revision surgery by cutting the stem.

## Discussions

Neuromuscular scoliosis treatment in children is a difficult challenge for an orthopaedic surgeon, the pathology evolving in complex spinal deformities even after reaching skeletal maturity, with a major impact on the quality of life, the morbidity, and mortality of these patients [**19**,**20**].

The Galveston pelvic fixation technique was introduced by Allen and Ferguson in 1984 [**14**] and used since then with great success and very good results, being a “gold standard” for neuromuscular scoliosis with pelvic obliquity correction even today. Luque segmental fixation with sublaminar wires introduced at every level from T2 to L5 offers excellent stability especially in demineralized and fragile vertebrae, frequently encountered in such patients [**21**-**27**]. Advances made in trans-pedicle screws/ hooks instrumentation led many authors to leave Luque-Galveston technique for the technique initially described by Cotrel-Dubousset for idiopathic scoliosis. Some surgeons are more familiar with this type of instrumentation, the surgery for idiopathic scoliosis being more frequent than for neuro-muscular ones. Still, conditions are not the same. As we emphasized, because most of these patients are non-walkers, the degree of osteoporosis for their vertebrae is important and often the screws are pulled out from the pedicles by the existent tensions forces. Also sacral or pelvic fixation with screws is more complicated, prolongs the operative time and augments intra-operative bleeding and other complications [**28**-**30**]. There are also hybrid fixation techniques described, which use Galveston pelvic fixation and lumbar screws. There is a biomechanical study conducted by Camp et al. [**31**] comparing iliosacral screwing with the Galveston technique. They showed that Galveston technique is the safer, the pulling force required for this type of fixation being much higher than in cases or iliosacral screws. More important, the pulling rate reported in the case of the screws techniques has been attributed to the poor quality of the existing bone for the neuromuscular patients. With the pelvic Galveston insertion, micro- mobility exists, but the rate of failures is low. The mobility chamber created in the months following the surgery disappears within the two following years. The same study showed a pulling rate of 44% for simple sacral screws, 28% for iliosacral screws, and 0% for Galveston. Studies that are more recent showed that there are cases of loose of the pelvic anchorage even with the Galveston technique [**17**]. Our study showed one patient with a complete migration of the rods into the pelvis. Happily, this patient did not need a whole revision of the construct, the fusion to the pelvis being satisfactory.

Our results showed that by using the Unit Rod technique, the correction is similar to that obtained by the Cotrel-Dubousset instrumentation concerning the Cobb angle and even better for pelvic obliquity. The stability in time is also the same. Intra and post-operative complications are minor. Shorter operating time and less laborious pelvic fixation diminishes intra-operative bleeding and complications [**18**]. As shown, the halo around the iliac stems does not affect stability, and disappears at around 2 years after surgery, the moment the spinal fusion is complete. In our study, neuro-muscular patients operated by the Unit Rod technique did not need an anterior approach [**11**,**12**,**32**]. There are studies [**12**] conducted on immature patients operated by the same technique (only posterior approach) before the closing of the triradiate cartilage, for whom no crankshaft effect occurred. Probably the rigidity of this construct prevents the possible rotation due to the remaining growth.

The primary fusion was obtained for most of our patients, the clinical outcomes were very positive [**33**,**34**], with no need of braces in their wheelchair, a good sagittal and frontal balance and a decreased rate of ischial pressure sores.

## Conclusion

Posterior spinal arthrodesis remains the intervention of choice for most kyphoscoliosis in neuro-muscular scoliosis. In most cases, arthrodesis allows non-usage of a brace, improves the installation of the patient in a wheelchair and his quality of life.

The Unit Rod technique is considered a technique of choice for the treatment of paediatric patients with neuro-muscular scoliosis. It is simple, rapid, without important intra- or post-operative complications, and it is considerably cheaper than other osteosynthesis systems. The radiological and clinical results are very good, similar to other techniques and it should be considered as the perfect instrumentation for medical health systems having financial problems.

Our study confirmed the significant functional and radiological improvements of the patients with a low risk of intra and postoperative complications. Our results were consistent with the ones already published in the literature for this technique.
